# Anxiety during transition from primary to secondary schools in neurodivergent children

**DOI:** 10.1002/jcv2.12262

**Published:** 2024-08-13

**Authors:** Vassilis Sideropoulos, Olympia Palikara, Elizabeth Burchell, Maria Ashworth, Jo Van Herwegen

**Affiliations:** ^1^ Department of Psychology and Human Development IOE UCL's Faculty of Education and Society University College London London UK; ^2^ Department of Education Studies University of Warwick Coventry UK

**Keywords:** anxiety, education, neurodiversity, school transition

## Abstract

Transition from primary to secondary school is an educational milestone that coincides with other key developmental changes associated with the onset of adolescence. Although previous studies have examined the impact of school transition on autistic students, no studies thus far have examined whether the impacts experienced are similar across different neurodivergent populations. In this paper, we examined how the transition to secondary school affects anxiety experienced by autistic children, children with Down syndrome (DS) and Williams syndrome (WS). Sixty‐one parents completed an online survey at two time‐points, answering questions about their neurodivergent child's anxiety, adjustment, well‐being, skills, and experiences of the transition to secondary school. The children themselves completed a short interview, which included completing a set of standardised measures with a researcher. Both neurodivergent children and parents expressed concerns about bullying and adjustment to new environments during transition from primary to secondary school. Although wide variability was found in our sample, no significant differences were revealed in the overall levels of parent‐reported anxiety pre‐ and post‐transition. However, different factors predicted anxiety during pre‐ and post‐school transition. The impact of these findings for theory and practice are discussed.

## Graphical abstract

No significant differences were observed in parent‐reported anxiety during the primary to secondary school transition in neurodivergent students.

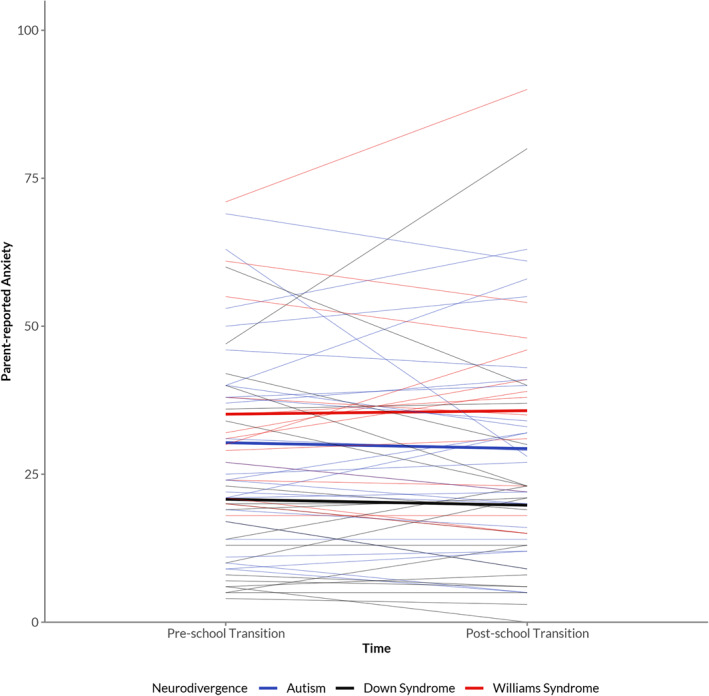

**Key Points**

- There is an urgent need to further understand how the transition to secondary school affects the mental health of neurodivergent children.
- While there is variability in parent‐reported anxiety amongst neurodivergent children, no significant differences are revealed between pre‐ and post‐secondary school transition.
- Different factors though are identified as predictors of parent‐reported anxiety pre‐ and post‐secondary school transition, indicating that the sources of stress and anxiety vary at different stages of the transition process.
- Both neurodivergent children and their parents expressed significant concerns about bullying and the adjustment to new environments during the school transition, highlighting the social and emotional challenges faced during this period.

## BACKGROUND

The transition from primary to secondary school is a key milestone in a young person's educational trajectory (Makin et al., 2017). This transition can provide opportunities for children to flourish and develop as individuals (Deieso & Fraser, 2019). However, it can also be a stressful and challenging period as children navigate multiple changes, including larger schools, new subjects, and more teachers (Humphrey & Ainscow, 2006). Clinicians, educators, and researchers are aware of the social, organisational, and emotional challenges associated with the transition from primary to secondary school and the impact they have on children's psychosocial wellbeing (Hughes et al., 2013; West et al., 2010). There is, however, an urgent need to further our understanding of how transition to secondary school affects the mental health of neurodivergent children, so we can identify key barriers and facilitators, as highlighted by the UK Department of Education's research priorities in 2024 (DfE, 2024). Learning more about the impact of transitions on neurodivergent children can inform efforts to promote inclusive practices in education.

A growing body of research has documented the vulnerability of neurodivergent children and their heightened risk of experiencing anxiety and stress during the transition from primary to secondary school (Dykens, 2000; Hughes et al., 2013; Mather & Ofiesh, 2007). Factors that contribute to this vulnerability are often associated with physical, pedagogical, and social changes, as well as interpersonal factors such as social understanding and sense of belonging (Galton & Morrison, 2000; Mumford & Birchwood, 2020).

Physical changes in the environment can be emotionally demanding for a child. During the transition from primary to secondary school, children often move to larger spaces with more than one building and multiple classrooms (Anderson et al., 2000). It has often been reported that these physical environmental changes increase the chances of distress to children as they risk getting lost (Coffey, 2013; Curson et al., 2019; Mizelle & Irvin, 2015) and that can increase anxiety (Karagiannopoulou, 1999). Pedagogical changes, such as being taught by several teachers as opposed to one teacher for all subjects, increased homework, and expectations of independence can negatively impact adjustment to the new environment and affect children's participation in the classroom (Anderson et al., 2000; Humphrey & Ainscow, 2006). Finally, social changes can create anxiety during school transitions. Peer relationships change during the primary to secondary school transition (Keay et al., 2015), causing fears about losing old friends, making new friends, and bullying (Curson et al., 2019; Gough Kenyon et al., 2020; Zeedyk et al., 2016). Based on previous research, neurodivergent pupils are more likely to be bullied across school years compared to neurotypical pupils (Maïano et al., 2016; Rose et al., 2010; Schroeder et al., 2014), which subsequently can have a significant impact on their mental health (Beckman et al., 2020).

Autistic children are at a greater risk of experiencing a negative transition to secondary school compared to neurotypical peers. Autism is a neurodivergence that is associated with differences in social communication, interaction, sensory processing, and patterns of repetitive behaviours from early on in development (American Psychiatric Association, 2013). Autism prevalence data suggests that, on average, there will be an autistic child in every classroom (Department of Health and Social Care, 2021). Autistic children are 4.5 times more likely to experience poorer mental health compared to neurotypical peers and other neurodivergent children (Dellapiazza et al., 2020; Schreck & Richdale, 2020), particularly generalised anxiety conditions (Kerns et al., 2020). Social anxiety is also common, as autistic children can find it increasingly difficult to navigate the wider socialisation demands of adolescence (Spain et al., 2018; White et al., 2009), leading to loneliness and difficulty in building peer networks (Locke et al., 2010).

Researchers have investigated the impact of transition to secondary school on autistic children's mental health. Mandy et al. (2016) reported high levels of psychopathology, maladjustment and bullying for autistic children at the end of primary school that persisted following the transition to secondary schools. Others have also discussed the challenges experienced by autistic children adjusting to and coping in their new secondary placements due to a lack of support during transitions (Dillon & Underwood, 2012; Makin et al., 2017; Tobin et al., 2012; Tso & Strnadová, 2016). Adjustment to the unfamiliar and unpredictable environment of secondary school, can increase anxiety and social pressure (Boulter et al., 2014; Nuske et al., 2019; Stack et al., 2021). Indeed, a review by Marsh et al. (2017) found that autistic children faced more challenges transitioning to school compared to neurotypical peers, and peers with other disabilities. Similarly, Bennett et al.’s review (2018) found that autistic, transition‐aged youth were at increased risk for mental health issues because of these challenges.

Children with Down syndrome (DS) or William syndrome (WS) are also at a higher risk for negative transition experiences due to the challenges they often experience in schools, such as bullying or maintaining friendships. DS is a neurodivergent condition occurring in about 1 in 800 live births and characterised by difficulties in language production, expressive speech development, memory, and mental health (Chapman & Hesketh, 2000; Fowler, 2009; Roberts et al., 2007; Tassé et al., 2016; van Gameren‐Oosterom et al., 2011; Will et al., 2019; Zampini & D’Odorico, 2013).

There are approximately 41,700 people with DS in England and Wales (Demography – DSMIG, 2022), but little is known about their experiences of transition to secondary placements. Researchers have identified some common and reoccurring patterns about their educational trajectories. For example, children with DS are usually sociable, engage easily in social activities and often make new friends easily (Hickey et al., 2012). However, their sociability could increase the chances of being bullied in attempts to socialise in new environments due to their social vulnerability (Fisher et al., 2013).

Children with DS are more likely to be educated in a mainstream school, in the UK and internationally, compared to other neurodivergent children (Freeman et al., 2016; Hargreaves et al., 2021; Van Herwegen et al., 2019). However, many pupils with DS (63%) move from mainstream primary schools to special secondary schools (Hargreaves et al., 2021). While there has been research into support systems and best practice in education for children with DS (Fidler & Nadel, 2007) as well as the benefits of parents and school staff planning together for school transitions (King et al., 2006; Lightfoot & Bond, 2013), there has been little research into mental health and anxiety during the transition to secondary school for pupils with DS. Some evidence suggests that children with DS score significantly lower on anxiety compared to their neurotypical peers (van Gameren‐Oosterom et al., 2011), while others report that patterns of anxiety in children with DS follow those of neurotypical children but show higher anxiety for issues around fear of strangers (Evans et al., 2005).

Similarly, there has been little research on the impact of transitions to secondary school for children with Williams syndrome (WS). WS is a neurodivergent condition which affects around 1 in 18,000 people in the UK (Williams Syndrome Foundation, 2022). WS is associated with similar cognitive profiles to children with DS, but children experience distinct difficulties in language and number development as well as spatial cognition (Van Herwegen, 2015). In terms of mental health, children with WS show higher levels of clinical anxiety, including general anxiety, separation anxiety, and fear of injury, with anxiety increasing as children get older (Glod et al., 2019; Little et al., 2013; Royston et al., 2018).

A recent parental survey has shown that pupils with WS are likely to transition from mainstream primary schools to special needs provision when they move to secondary schools (Van Herwegen et al., 2018). However, no studies have examined the process of transitioning from primary to secondary school or the impact of that process on anxiety in pupils with WS directly (Palikara et al., 2018). As children with WS often experience high anxiety in general and prefer structured routines (Palikara et al., 2018; Royston et al., 2018), it can be predicted that they are likely to be impacted negatively by transition to secondary school and experience elevated levels of anxiety. Correspondingly, despite their overly outgoing social nature (Dykens, 2003), children with WS struggle to obtain meaningful friendships with peers (Klein‐Tasman et al., 2011) and a transition to a new school could possibly trigger anxiety.

Altogether, research suggests that transition from primary to secondary school can be challenging for neurodivergent children, and that it can increase anxiety levels (Anderson et al., 2000; Coffey, 2013; Curson et al., 2019). However, it is still unclear whether there are overlaps or differences in the transition experiences amongst neurodivergent children. For example, neurodivergent pupils that experience elevated levels of anxiety (e.g. autistic children), may be impacted more than those who experience less anxiety (e.g. children with DS), during transitions (Rous et al., 2007). Transition to secondary school not only affects neurodivergent children, but their parents and teachers too (Nuske et al., 2018). Parents often worry for their children's wellbeing and adjustment to school, and they feel alienated in school discussions (Fontil et al., 2020; Tso & Strnadová, 2016). For example, parents often are not aware of changes that occur in their child's school life and hence cannot provide full support and be understanding towards their child. Research indicates that there is a similar lack of guidance, resources, and training for teachers on how best support to neurodiverse children during school transition (Fontil et al., 2020) as well as how to promote inclusion (Tso & Strnadová, 2016).

There is a plethora of factors that need to be considered to gain a comprehensive understanding of school transitions, such as investigating the experience at an individual‐, family‐, school‐ and systemic‐level, and gathering key stakeholders' perspectives (i.e. the child‐, parent‐ and teacher). The existence of those problems has been discussed in the neurotypical literature and most research suggests that distress and negative emotions diminish as the child adjusts to their new school environment (Makin et al., 2017). This also seems to be the case for autistic children (Mandy et al., 2016). However, it is important to understand whether stressful school transitions represent a common universal experience amongst neurodivergent children.

Drawing upon the findings from previous studies that have mainly looked at the impact of school transitions in specific neurodivergent populations, the current study aimed to identify clusters of overlaps and differences in anxiety pre‐ and post‐school transition amongst neurodivergent children, namely autistic children, and children with WS and DS. Comparing the experiences of children with three different neurodivergences will elucidate the similarities and differences in the transition from primary to secondary school between autistic children (a population which has been researched extensively), and children with DS and WS (two populations whose transition experience has been under‐researched). This study includes views from both neurodivergent children and parents across the transition process to build a more holistic understanding of anxiety during this educational milestone, by addressing the following research questions:

1. What is the impact of school transition on anxiety and how does the impact differ between neurodivergences?
2. What factors predict the effect of school transition on anxiety scores and how do these factors differ across neurodivergent children?
3. What do parents and children report about the outcomes of the transition from primary to secondary school process, in terms of bullying, belonging (i.e. having friends, liking the new school) and being settled?

For the first research question, it was predicted that autistic children and children with WS would experience higher levels of anxiety pre‐ and post‐school transition (Makin et al., 2017) when compared to children with DS (Evans et al., 2005). Based on previous literature and our clinical experience, an exploratory approach is taken for the second research question considering no previous research has identified any predictors.

Finally, transition periods are a challenging time for children, thus it was predicted that parents would report higher levels of concerns and worries about their child's psychosocial wellbeing, especially around bullying and being settled in the school (Nuske et al., 2018). In terms of children's reports, since there is little research that investigates the concerns of children with DS and WS, we expected autistic children to report higher frequency of bullying and lower scores for having friends and school liking and higher levels of school refusal (i.e. being settled) compared to those with DS and WS. We predicted that levels would change as a result of school transition (i.e. more bullying, less friendships, higher level of school refusal, and lower levels of school liking).

## METHOD

The data presented in this paper are part of a larger study on transitions to secondary schools for neurodivergent children. In this paper we focus on the quantitative mental health outcomes reported by parents and their neurodivergent children.

### Participants

Participants were neurodivergent children living in the UK with a formal diagnosis of either autism, DS or WS, who were set to experience a transition to secondary school (i.e. a transition to a different school or a transition to a different school‐site or new building), and one parent per neurodivergent child participant. The final participant sample for this study included child‐parent dyads who completed all the measures pre‐ and post‐transition.

Families were recruited nationally through advertising across various forums: special needs support groups, charity foundations including the Williams Syndrome Foundation UK and Downs Syndrome Association, social media campaigns, and approaching schools directly. Thus, families could have accessed the survey through several routes. An overview of the participants can be found in Tables 1 and 2.

**TABLE 1:** Sociodemographic characteristics of neurodivergent children.

Characteristic	Neurodivergence			Total (*n* = 61)
	Autism (*n* = 25)	Down syndrome (*n* = 21)	Williams syndrome (*n* = 15)	
Age in months, *M* (*SD*)	135.9 (*SD* = 3.8)	139.1 (*SD* = 5.1)	134.1 (*SD* = 4.6)	
Gender				
Female	7	8	8	23
Male	18	13	7	38
School type^a^				
Mainstream	22	15	6	43
Special needs	2	4	8	14
Mixed	1	2	1	4
Other	‐	‐	‐	‐
School type^b^				
Mainstream	17	9	1	27
Special needs	3	12	13	28
Mixed	3	‐	1	4
Other	2	‐	‐	2
EHCP pre‐school transition				
Yes	9	21	14	44
No	13	‐	‐	13
Missing responses	3	‐	1	4
EHCP post‐school transition				
Yes	14	21	15	46
No	11	‐	‐	11
Missing response	‐	‐	‐	‐
Co‐morbidity				
Yes	17	5	4	26
No	8	16	11	35

Abbreviation: EHCP, Education Health and Care Plan.

^a^ Pre‐school transition.

^b^ Post‐school transition.

**TABLE 2:** Socio‐demographic characteristics of parents of neurodivergent children.

Characteristic	Neurodivergence			Total (*n* = 61)
	Autism (*n* = 25)	Down syndrome (*n* = 21)	Williams syndrome (*n* = 15)	
Age group				
31–40 years	5	1	7	13
41–50 years	14	17	6	37
51–59 years	6	3	2	11
Ethnicity or race				
Asian	‐	1	1	2
Black or African American	‐	1	1	2
White or European American	22	19	13	54
Mixed	2	‐	‐	2
Other^a^	1	‐	‐	1
Gender				
Female	23	21	12	56
Male	2	‐	3	5
Highest level of Education^b^				
No formal education	‐	‐	1	1
GCSE or equivalent	2	2	3	7
A‐level	3	4	2	9
Vocational	1	1	‐	2
Graduate	8	7	6	21
Post‐graduate	10	7	3	20
Current employment status^b^				
Full time employed	7	2	6	15
Part time employed	11	12	5	28
Volunteer	‐	1	1	2
Prime homemaker	7	6	2	15
Unemployed	‐	‐	‐	‐
Student	‐	‐	1	1

^a^ The ‘other’ classification was composed of participants who self‐identified as Latina, Filipina, or multiracial.

^b^ Pre‐school transition.

### Procedure and materials

Parents completed an online survey hosted in Qualtrics. Neurodivergent children engaged in an online interaction with the researcher via the video conferencing software (Zoom) to (a) complete the related cognitive assessments, and (b) answer the survey questions which were read out by the researcher (with the assistance of visual aids). This approach can be cost‐effective and inclusive for those populations (Ashworth et al., 2021), but the validity of the results should always be considered as a recent systematic review discusses the limits of tele‐assessment of cognitive functions in children (Ruffini et al., 2022).

All participants completed their respective measures at two time‐points, pre‐ and post‐school transition. Specifically, data was collected when the children were attending the last year of their primary school (Time 1) and then again during the first year of secondary school (Time 2). Data was collected from 2018 up to December 2019 and thus was not impacted by the COVID‐19 pandemic.

### Measures

#### Parent measures

##### Spence Children's Anxiety Scale

The Spence Children's Anxiety Scale (SCAS‐P) (Spence, 1999) is a standardised measure and was used to measure children's anxiety (*α* = .87, indicating high levels of reliability). The SCAS‐P consists of 38 items that a child might worry about (e.g. my child worries about things) divided into 6 subscales: generalised anxiety, panic, separation, physical injury, social phobia, and obsessive‐compulsive condition. Parents rated their child on these items on a 4‐point Likert scale from 0 (never) to 3 (always) scale. The higher score reflects greater anxiety on each subscale and the overall total score. For the purpose of this study, we used the overall total score of the SCAS‐P. For timepoint 1, only 2 autistic children and 2 children with WS scored above clinical cut‐off (total score above 60). For timepoint 2, there were only 2 autistic children, 1 child with DS and 1child with WS that scored above clinical cut‐off.

##### The Strengths and Difficulties Questionnaire

The Strengths and Difficulties Questionnaire (SDQ) (Goodman, 1997) is a standardised measure and was used to measure children's internalising and externalising behaviours from a parental perspective. The SDQ consists of 25 items rated on a 3‐point Likert scale from 1 (*Not True*) to 3 (*Certainly True*) and is divided into 5 subscales: emotional symptoms, conduct problems, hyperactivity, peer relationships problems, and prosocial behaviour. These can be later transformed to internalising (the sum of emotional and peer problems scales) and externalising (the sum of conduct and hyperactivity scales) scores. The higher scores reflect greater levels of behavioural difficulties.

Both the SCAS‐P and SDQ were completed at Time 1 and Time 2 by the parents.

#### Child measures

A set of standardised measures was employed to assess various aspects of cognitive abilities.

##### British Picture Vocabulary Scale

The British Picture Vocabulary Scale (BPVS) (Dunn and Dunn (2009)) is a standardised task‐based measure for receptive language abilities. Its administration involves the examiner saying a word and the examinee responding by selecting a picture from four options that best illustrates the meaning of the word. The scores are calculated based on the number of correct responses. The higher scores reflect better receptive vocabulary abilities.

##### Raven's Progressive Coloured Matrices

Raven's Progressive Coloured Matrices (RPCM) (Raven & Court, 1998) is a standardised task‐based measure. The test consists of coloured patterns with a piece missing; the task is to pick the correct missing piece from six options. The scores are calculated based on the number of correct responses, with higher scores indicating higher fluid intelligence and abstract reasoning.

##### Vineland Adaptive Behaviour Scales

Vineland Adaptive Behaviour Scales (VABS) (Sparrow & Cicchetti, 1989) is an interview‐based measure, with scores assigned by the assessors. We focused on the non‐adaptive subscale as it measures behaviours like excessive worry, avoidance, or difficulty coping with change, that are often apparent in transition periods. The higher scores indicate greater independence and proficiency in adaptive behaviours.

##### Social Responsiveness Scale

Social Responsiveness Scale (SRS) (Constantino & Gruber, 2012) was used to assess social skills. SRS consists of 65 items where the participant has to rate themselves from 1 (*Not True*) to 4 (*Almost Always True*). The scale consists of five domains: Social Awareness, Social Cognition, Social Communication, Social Motivation, and Restricted Interests and Repetitive Behaviour. In our study we used to total score to account for all aspects. The higher scores indicating greater social difficulties.

The BPVS, RPCM, and SRS were assessed only at Time 1 as there was not a substantial interval between timepoints to account for possible changes over time. VABS was assessed at both Time 1 and Time 2. In addition, due to the small sample size of our study, we computed a new composite variable for cognitive abilities to avoid overfitting the model. This new variable combined the BPVS and Ravens scores. Table 3 presents the mean scores across all measures, including the newly computed variable. Supporting Information S1 provide full ANOVA models and post‐hoc analyses.

**TABLE 3:** Mean, standard deviation, and differences amongst the standardised measures.

Timepoint	Measure	Neurodivergence	*N*	Missing	Mean	Std. deviation	Min	Max
Pre‐school transition	Anxiety	Autism	25	0	30.320	16.698	9	69
		Down syndrome	21	0	20.762	16.229	4	60
		Williams syndrome	14	1	35.143	16.081	18	71
	Internalising	Autism^a^	25	0	11.400	3.697	4	19
		Down syndrome^b^	21	0	4.714	2.572	0	10
		Williams syndrome	15	0	9.267	2.685	4	13
	Externalising^c^	Autism	25	0	10.000	3.873	3	17
		Down syndrome	21	0	7.619	3.074	1	13
		Williams syndrome	15	0	9.867	2.722	5	15
	Fluid intelligence	Autism^d^	24	1	31.833	5.662	9	36
		Down syndrome^e^	20	1	13.200	5.033	4	27
		Williams syndrome	15	0	15.133	4.324	8	23
	Receptive language abilities	Autism^f^	24	1	136.000	25.259	32	160
		Down syndrome^g^	20	1	73.900	23.074	24	107
		Williams syndrome	15	0	90.067	14.621	64	110
	Social skills	Autism^h^	25	0	88.680	25.677	51	151
		Down syndrome^i^	21	0	69.524	24.847	33	138
		Williams syndrome	15	0	88.200	20.844	59	139
	Non‐adaptive behaviours^j^	Autism	25	0	24.960	9.103	10	41
		Down syndrome	21	0	21.905	10.483	7	44
		Williams syndrome	15	0	24.867	7.873	11	39
	Cognitive abilities	Autism^k^	24	1	167.833	30.249	41	195
		Down syndrome^l^	20	1	87.100	26.338	32	134
		Williams syndrome	15	0	105.200	17.301	75	128
Post‐school transition	Anxiety	Autism	25	0	29.320	17.082	5	63
		Down syndrome	21	0	19.762	17.481	0	80
		Williams syndrome	15	0	35.733	19.536	15	90
	Internalising	Autism^m^	25	0	10.520	3.874	2	17
		Down syndrome^n^	21	0	4.476	2.768	1	12
		Williams syndrome	15	0	9.667	2.895	4	14
	Externalising^o^	Autism	25	0	9.280	3.506	3	18
		Down syndrome	21	0	6.952	2.312	3	10
		Williams syndrome	15	0	10.467	1.727	6	13
	Non‐adaptive behaviours^p^	Autism	25	0	24.720	9.787	9	50
		Down syndrome	21	0	18.238	8.723	4	41
		Williams syndrome	15	0	25.400	5.539	14	35

^a^ Significantly higher than Down syndrome (*p* < .001), but not Williams syndrome (*p* = 1).

^b^ Significantly lower Williams syndrome (*p* < .001).

^c^ No significant difference between the groups—see Supporting Information S1 for all the *p*‐values.

^d^ Significantly higher than Down syndrome (*p* < .001) and Williams syndrome (*p* < .002).

^e^ No significant difference between Down syndrome and Williams syndrome (*p* = .827).

^f^ Significantly higher than Down syndrome (*p* < .001) and Williams syndrome (*p* < .001).

^g^ No significant difference between Down syndrome and Williams syndrome (*p* = .14).

^h^ Significantly higher than Down syndrome (*p* = .03), but no difference between WS (*p* = 1).

^i^ No significant difference between Down syndrome and Williams syndrome (*p* = .08).

^j^ No significant difference between groups (*p* = .129).

^k^ Significantly higher than Down syndrome (*p* < .001) and Williams syndrome (*p* < .001).

^l^ No significant difference between Down syndrome and Williams syndrome (*p* = .143).

^m^ Significantly higher than Down syndrome (*p* < .001) but now Williams syndrome (*p* = 1).

^n^ Significantly lower than Williams syndrome (*p* < .001).

^o^ No significant difference amongst the groups—see Supporting Information S1 for the *p* values.

^p^ No significant difference amongst groups (*p* = .129).

#### Parent and child measures

A set of questions about the school experiences were asked to both parents and children after they had moved to secondary school (T2). These questions were either binary (yes/no) or had an extra option (Prefer not to disclose/Do not know/No change). Children were asked these questions verbally whereas parents completed these online. These questions are presented later in Table 5 and covered school belonging (i.e. having friends or being bullied), school refusal, liking school and being settled.

### Statistical analysis

To identify predictors of anxiety during the transition to secondary school, we computed a series of linear regression models for both pre‐ and post‐school transition. Then, a repeated measures ANOVA was also computed to compare anxiety for each group (3) between pre‐ and post‐school (2) transition. Partial eta squared ($\eta_{p}^{2}$) effect sizes are also reported. Planned post‐hoc comparisons were used to compare group differences using Bonferroni corrections. Finally, tests of associations 2 (yes/no) × 3 (Autism, DS, WS) were computed to investigate the parental and child reports on several aspects.

It is important to note that we have identified outliers in our sample which we decided to include in our analyses for statistical power and to account for the variability within our neurodivergent sample. All analyses were computed using SPSS v.28.

## RESULTS

### Impact of primary to secondary school transition on anxiety

A descriptive breakdown of anxiety showed that 11 out of 25 (44%) autistic children scored above the clinical cut‐off for anxiety during the pre‐school transition timepoint. In contrast, only 6 out of 21 (28.57%) children with DS did, and 8 out of 14^1^ (57.14%) children with Williams Syndrome. For the post‐school transition, 11 out of 25 (44%) autistic children scored above the clinical cut‐off point, 4 out of 21 (19.05%) children with DS, and 9 out of 15 (60%) with Williams Syndrome.

**FIGURE 1:**
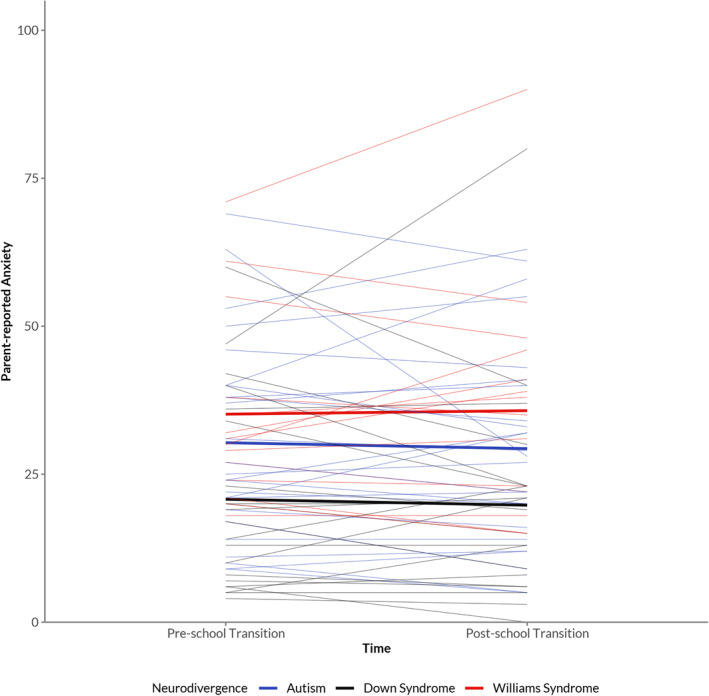
Parent‐reported anxiety during primary to secondary school transition in neurodivergent students. Thick lines represent the average anxiety levels over time for each group. Thin lines represent the individual trajectories of anxiety levels as reported by their parents.

A repeated measures ANOVA was conducted to compare the effect of time (pre‐ and post‐school transition/T1 and T2) on children's anxiety scores, across our three neurodiverse groups. There was no significant effect of time, *F*(1, 57) = 0.008, *p =* .928, $\eta_{p}^{2}$ = 0.000, or interaction between time and group, *F*(2, 57) = 0.385, *p =* .682, $\eta_{p}^{2}$ = 0.013. However, there was a significant effect of group, *F*(2, 57) = 4.126, *p* = .021, $\eta_{p}^{2}$ = 0.126. SCAS‐P scores were significantly higher in the WS group compared to the DS group (*p* = .023). There were no differences between the autism and WS group (*p* = .802) nor the autism and the DS group (*p* = .163). By looking at the means, autistic children scored almost the same pre‐ (*M* = 30.3) and post‐school transition (*M* = 29.3). The same patterns for children with DS (pre‐transition *M* = 20.8 and *M* = 19.8 post‐school transition) and for children with WS (pre‐transition *M* = 35.1 and *M* = 36.8 post‐school transition). Figure 1 visualises the scores of pre‐ and post‐school transition for all three groups.

### Predictors of impact of school transition on anxiety (regressions per group)

Six multiple linear regressions were computed for each of the three neurodiverse groups to predict Anxiety (Total SCAS‐P) from the following variables: cognitive abilities (BPVS and RCPM), social skills (SRS), internalising and externalising behaviours (SQD) and non‐adaptive behaviour (VAB) for both pre‐ and post‐transition. To maximise the value of the data we have available, we excluded cases using a listwise deletion. The reported models, along with the coefficients, are presented in Table 4.

**TABLE 4:** Multiple linear regression summaries and coefficients.

Model	Unstandardised	Standard error	Standardised	*t*	*p*	VS‐MPR^a^	95% CI	
							Lower	Upper
Model 1: Parent‐reported anxiety for autistic children—Pre‐school transition								
Model 1 summary: *F*(5, 18) = 3.201, *p* < .05, *R* ^2^ = .471 and *R* ^2^ adjusted = .324								
Coefficients								
Internalising	2.367	0.973	0.526	2.433	0.026*	3.920	0.323	4.410
Externalising	−1.237	0.997	−0.289	−1.240	0.231	1.087	−3.332	0.858
Social abilities	−0.148	0.156	−0.228	−0.947	0.356	1.000	−0.475	0.180
Cognitive abilities	−0.010	0.106	−0.018	−0.093	0.927	1.000	−0.232	0.213
Non‐adaptive behaviours	5.259	2.654	0.563	1.982	0.063	2.112	−0.316	10.834
Model 2: Parent‐reported anxiety for autistic children—Post‐school transition								
Model 2 summary: *F*(5, 18) = 7.469, *p* < .001, *R* ^2^ = .675 and *R* ^2^ adjusted = .584								
Coefficients								
Internalising	1.329	1.063	0.311	1.250	0.227	1.093	−0.904	3.562
Externalising	−1.690	1.224	−0.356	−1.381	0.184	1.180	−4.261	0.882
Social abilities	−0.124	0.108	−0.193	−1.150	0.265	1.045	−0.352	0.103
Cognitive abilities	−0.023	0.082	−0.040	−0.275	0.786	1.000	−0.195	0.150
Non‐adaptive behaviours	7.595	3.213	0.819	2.364	0.030*	3.537	0.845	14.345
Model 3: Parent‐reported anxiety for DS children—Pre‐school transition								
Model 3 summary: *F*(5, 14) = 5.117, *p* < .05, *R* ^2^ = .649 and *R* ^2^ adjusted = .524								
Coefficients								
Internalising	3.875	1.142	0.627	3.394	0.004**	15.513	1.427	6.324
Externalising	0.309	1.050	0.057	0.294	0.773	1.000	−1.942	2.560
Social abilities	−0.252	0.148	−0.389	−1.704	0.111	1.511	−0.568	0.065
Cognitive abilities	0.095	0.104	0.153	0.907	0.380	1.000	−0.129	0.319
Non‐adaptive behaviours	3.299	1.832	0.430	1.801	0.093	1.662	−0.631	7.229
Model 4: Parent‐reported anxiety for DS children—Post‐school transition								
Model 4 summary: *F*(5, 14) = 1.453, *p* > .05, *R* ^2^ = .342 and *R* ^2^ adjusted = .107								
Coefficients								
Internalising	3.514	1.783	0.599	1.971	0.069	1.997	−0.310	7.337
Externalising	−0.543	2.377	−0.075	−0.229	0.823	1.000	−5.642	4.556
Social abilities	0.114	0.200	0.176	0.570	0.578	1.000	−0.315	0.543
Cognitive abilities	0.114	0.152	0.184	0.749	0.466	1.000	−0.212	0.439
Non‐adaptive behaviours	−1.761	3.359	−0.197	−0.524	0.608	1.000	−8.965	5.443
Model 5: Parent‐reported anxiety for WS children—Pre‐school transition								
Model 5 summary: *F*(5, 8) = 4.006, *p* < .05, *R* ^2^ = .715 and *R* ^2^ adjusted = .536								
Coefficients								
Internalising	−0.026	1.640	−0.004	−0.016	0.988	1.000	−3.809	3.757
Externalising	0.326	1.208	0.057	0.270	0.794	1.000	−2.460	3.113
Social abilities	0.734	0.293	0.907	2.506	0.037*	3.038	0.059	1.410
Cognitive abilities	−0.246	0.186	−0.266	−1.324	0.222	1.101	−0.674	0.182
Non‐adaptive behaviours	−2.315	2.865	−0.236	−0.808	0.442	1.000	−8.920	4.291
Model 6: Parent‐reported anxiety for WS children—Post‐school transition								
Model 6 summary: *F*(5, 8) = 4.650, *p* < .05, *R* ^2^ = .744 and *R* ^2^ adjusted = .584								
Coefficients								
Internalising	1.278	2.039	0.239	0.627	0.548	1.000	−3.424	5.979
Externalising	−2.649	2.373	−0.286	−1.116	0.297	1.020	−8.121	2.823
Social abilities	0.659	0.229	0.814	2.880	0.021*	4.616	0.131	1.186
Cognitive abilities	−0.018	0.241	−0.019	−0.075	0.942	1.000	−0.573	0.537
Non‐adaptive behaviours	−2.252	3.417	−0.177	−0.659	0.528	1.000	−10.131	5.627

*Note:* Significance levels: *p < 0.05, **p < 0.01.

^a^ Vovk–Sellke maximum p‐ratio: based on the p‐value, the maximum possible odds in favour of *H*₁ over *H*₀ equals 1/(−e p log(p)) for p ≤ .37 (Sellke et al., 2001).

### Autism group

Model 1 revealed that autistic children, as reported by parents, who exhibited more internalising behaviours (parent‐reported: *b* = 2.176, *β* = .609) were associated with higher levels of parent‐reported anxiety during the preschool transition period. On the other hand, Model 2 showed that parents of autistic children who reported increased non‐adaptive behaviours pre‐school transition also reported more anxiety in the post‐transition period (*b* = 7.595, *β* = .819).

### DS group

In Model 3, it was found that children with DS, as reported by parents, who exhibited more internalised behaviours (parent‐reported: *b* = 3.875, *β* = .627) during the preschool transition period also showed increased levels of parent‐reported anxiety. However, in Model 4, none of the factors predicted parent‐reported anxiety during the post‐school transition for children with DS.

### WS group

Both Model 5 and Model 6 revealed that children with WS who self‐reported more social challenges, experienced higher levels of parent‐reported anxiety during both the pre‐school (*b* = 0.734, *β* = .293) and post‐school transition periods (*b* = 0.659, *β* = .229).

### Parental and child reports on outcomes related to transition process

Across all three groups, most children reported that they liked their new school, *χ* ^2^(4, 55) = 5.28, *p* = .26, and that they had made friends, *χ* ^2^(2, 57) = 2.72, *p* = .26 (see Table 5 for detail breakdown per neurodivergence). However, children with WS stated significantly higher incidence of being teased or bullied, compared to those with DS or autistic children; *χ* ^2^(2, 57) = 10.04, *p* < 6.59e‐3.

**TABLE 5:** Child and parent‐reported concerns about transitions for neurodivergent children.

Transition concerns	Neurodivergence		
	Autism (*n* = 25)	Down syndrome (*n* = 21)	Williams syndrome (*n* = 15)
	*N* (%)	*N* (%)	*N* (%)
Reported by children			
I like my new school			
Yes	19 (34.55%)	18 (32.73%)	11 (20.00%)
No	4 (7.27%)	‐	2 (3.64%)
Don't know	‐	1 (1.82%)	‐
Did you make new friends?			
Yes	20 (35.09%)	19 (33.33%)	14 (24.56)
No	3 (5.26%)	‐	1 (1.75)
I get teased/bullied at school			
Yes	11 (19.30%)	2 (2.51%)	9 (15.79%)
No	12 (21.05%)	17 (29.82%)	6 (10.53%)
Reported by parents			
Have you noticed a change in your child since they moved to secondary school?			
Yes, positive change	8 (13.56%)	4 (6.78%)	5 (8.47%)
Yes, negative change	15 (25.42%)	17 (28.81%)	10 (16.95%)
No change	‐	‐	‐
Has your child ever refused to go to their new school or tried to get out of going?			
Yes	9 (14.75%)	5 (8.20%)	3 (4.92%)
No	16 (26.23%)	16 (26.23%)	12 (19.67%)
Has your child had any problems with bullying (e.g. called nasty names, teased, physically hurt) at their new school?			
Yes	11 (20.8%)	3 (5.7%)	7 (13.2%)
No	9 (17%)	15 (28.30%)	8 (15.01%)
Adjustment to new school			
How well has your child settled into their new school?	*M* = 6.32 (*SD* = 2.51)	*M* = 8.52 (*SD* = 1.03)	*M* = 8.00 (*SD* = 1.96)
On a scale from 0 (not well) to 10 (very well)			

Parents reported that they had noticed a negative change in their child since the transition for about 19%–32% of the children (see Table 5). There were no significant group differences, *χ* ^2^(2, 59) = 1.53, *p* = .47. There was also no significant difference between the three groups for school refusal: *χ* ^2^(2) = 1.46, *p* = .48. In terms of their child being bullied, parental reports did not vary from what the children reported, with significant differences between the three groups with the parents of autistic children and of children with WS reporting more problems with bullying: *χ* ^2^(2, 53) = 6.25, *p* = .04 at post‐school transition.

There were group differences for how well the children had settled into their new school as reported by the parents, with children in the autism group scoring significantly lower than those with DS or WS; *F*(2, 57) = 7.72, *p* < 1.08. Bonferroni post‐hoc tests showed significant differences between autism and DS groups (*p* = .001), autism and WS groups (*p* = .04), but no significant difference between DS and WS (*p* = 1.00).

## DISCUSSION

To the best of our knowledge, this is the first study that examines how transition from primary to secondary school affected the anxiety levels of neurodivergent children and attempts to identify clusters of overlap and difference amongst them. Primarily, we explored parent‐reported anxiety levels for pre‐ and post‐school transition, then we examined both parent‐reported and child‐reported predictors of these anxiety levels. Finally, we examined parental and child reports about belonging and being settled within the school they attended after transition. This study not only provides evidence to better understand the variability within each group, but it also identifies clusters of overlap which helps us understand the unique profiles of specific neurodiverse groups when thinking about for how to support the transition from primary to secondary schools for those with different neurodivergences.

### Impact of primary to secondary school transition on anxiety

For our first hypothesis, we expected autistic children and children with WS to exhibit higher levels of self‐reported anxiety compared to children with DS during the pre‐ and post‐school transition times. As predicted, the parent‐reported anxiety data revealed that children with WS scored significantly higher compared to those with DS. However, there were no differences between autistic children and children with WS nor or children those with DS. Despite the mean differences amongst neurodivergent children, there was no difference between their pre‐ and post‐times of the parent‐reported anxiety scores. This finding replicates findings from self‐reports of anxiety of autistic children, that there was no difference in anxiety between pre‐ and post‐transition (Mandy et al., 2016).

Overall, this analysis revealed that some autistic children were perceived by their parents as experiencing high levels of anxiety. In the educational literature, high levels of anxiety in the autistic population were often associated with a more challenging transition to secondary school (Kerns et al., 2020; Nuske et al., 2018, 2019). Considering that the parent‐reported anxiety in our study, matches the self‐reports of autistic children from the previous literature, we could argue that similarly that is the case for some children with WS (Palikara et al., 2018; Royston et al., 2018) and with DS as our descriptive statistics showed. For instance, for some children with WS and DS parents‐reported high levels of anxiety before and after transition. However, these high levels were only observed to a specific number of participants. Further research is needed in this area to identify which participants might be at lower or higher risk for increased anxiety during this key educational milestone, through the use of self‐reports.

### Predictors of anxiety for pre‐ and post‐school transition

Higher parent‐reported anxiety in autistic children was associated with increased scores in parent‐reported internalised behaviours during pre‐transition timepoint. However, the parent‐reported anxiety of autistic children was only associated with the self‐reported non‐adaptive behaviours in the post‐transition timepoint. Internalising behaviours, such as social withdrawal, peer difficulties, and inhibition of feelings, are recognised precursors to anxiety during school transitions (Storch et al., 2012; White et al., 2015). Moreover, autistic children frequently engage in non‐adaptive behaviours (Hartley et al., 2008) and these behaviours are closely associate with anxiety (Rodgers et al., 2012). In summary, our findings could suggest that some autistic children experience high anxiety before and after the transition to secondary school, and that their experience of anxiety may be influenced by how they handle their emotions. However, these findings need to be considered with their limitations (e.g. parent‐reported scales) and researchers need to study anxiety in these populations using self‐reports.

For the children with DS, only parent‐reported internalising behaviours were associated with higher levels of parent‐reported anxiety in pre‐school transition, and no factor predicted anxiety in the post‐school transition period. Overall, previous research documents the high rates of emotional and behavioural problems in individuals with DS (Nærland et al., 2017) which are the two key aspects measured by our parent‐reported scale for internalising behaviours and potentially could lead to higher anxiety.

For children with WS, only self‐reported social challenges were associated with higher levels of parent‐reported anxiety at pre‐ and post‐school transition. This result is consistent with existing research. For example, teachers often report that children with WS show social reciprocity difficulties around social cognition, communication and engagement which could also be due to the new school environment (Klein‐Tasman et al., 2011). In addition, despite their strong drive for socialisation, children with WS find it more challenging to socialise and form meaningful relationships as they get older (Royston et al., 2019). Subsequently, their low social skills are also the reason for having increased risk for social isolation, bullying and unsteady relationships (Jawaid et al., 2012).

Overall, our data revealed a cluster of overlap between autistic children and children with DS during the pre‐school transition time as both engaged in internalising behaviours, as reported by parents. Such evidence suggests that these children experiencing anxiety around the time of transition, without vocalising their concerns. This is significant for practice as teachers can provide similar support to autistic and DS children. While these factors were not significant in children with WS, our data suggest that those children could benefit from a similar approach to autistic and DS children as they also struggle with social challenges during transition to secondary school, such as social communication and engagement. Anxiety in children with WS often is often triggered by phobias and uncertainty (Royston et al., 2021), whilst anxiety in autistic children is often due to difficult social situations and sensory environments (McDonnell et al., 2015). However, it appears that during transition to secondary school the support levels required are similar.

### Child and parental reports on outcomes related to the transition process

Autistic children scored higher on negative transition outcomes, as they were less likely to be settled into their new school and reported bullying, as predicted. Previous literature suggests that this is common for autistic children as they are more likely to be anxious and stressed about several school‐related aspects (Kerns et al., 2020; Nuske et al., 2018, 2019) during the transition to secondary school. Our data also showed that there were no differences in settlement between children with DS and WS. The lack of difference between the WS and DS group could be rooted in the fact that both groups are very social, despite their fears and struggles. Similarly, there is no difference between children with DS and WS for reports around liking their new school, making new friends, or settling in the new school. These findings highlight the clusters of difference between autistic children and children with DS and WS, but also the clusters of overlap between children with DS and WS when settling into the new environment.

Autistic children also reported being teased or bullied in their secondary provision more compared to children with DS. However, children with WS reported more bullying than autistic children. Parental reports echoed the findings for bullying. These findings replicate previous research which has shown that parents of autistic children tend to report more worries about problems with bullying (Fontil et al., 2020; Nuske et al., 2018). Considering the lack of research on transition experiences in WS and DS (Palikara et al., 2018), our findings highlight that some neurodivergent pupils are at more risk of bullying than others. Nonetheless, further research in this area is required to fully understand the bullying experiences and its impact on neurodivergent pupils. Finally, our study provides evidence that bullying percentages are indeed high in some neurodivergent populations (Maïano et al., 2016; Rose et al., 2010; Schroeder et al., 2014).

Whilst in the neurotypical literature it has been found that issues related to bullying and belonging can lead to high school absence (Gubbels et al., 2019), there was no difference between groups on school refusal despite the bullying problems experienced by the neurodivergent children. Still, parents reported a noticeable negative change in the children's demeanour since the transition across the three groups. However, more research with bigger sample sizes is needed to account for individual variability in the neurodiverse populations.

### Limitations and future studies

One important limitation of the study is the small sample size, which is a common and recurring issue in the field of neurodiversity. However, it is important to stress that the children recruited in the study are from a narrow age range, which allows better insight into the variability within a particular age group but limits the size of the group. In addition, for children with WS, we recruited about half the population for that age in the UK, according to the number of people for that age range registered with the Williams Syndrome Foundation.

Another limitation is also the accurate representation of the parents; there is a possibility that our study attracted only those parents who are not satisfied with the school transitions procedures or the performance of their child in the school. As parents completed the SCAS‐P and SDQ, there might be a bias as to how they perceived their children's mental health and internalising and externalising behaviours. However, research has shown that parents are usually very good at estimating their child's abilities (Chandler et al., 2016). Finally, in this study we assessed neurodivergent children within the first term in their secondary school; and problems might not become evident until later. For example, lack of support or poor academic achievement may affect neurodivergent children in the longer term (Richter et al., 2019). Hence, the lack of difference across several factors might be due to the short interval between the pre‐ and post‐school transition measures.

Future research should focus on longitudinal data to understand the mental health of neurodivergent children as there are many different factors that can impact their psychosocial wellbeing through their life course. At the time of the first assessment, children were already aware that a transition would happen and thus anxiety might already have been higher. Assessing anxiety at different time points across the life span would allow a further understanding of how unique the anxiety patterns during school transition are, compared to other times of transition.

## IMPACT AND CONCLUSION

The present study provides a deeper insight into the transition from primary to secondary school for neurodivergent children. To begin with, it showed that all neurodivergent children reported difficulties related to the transition, but experiences varied, for instance more autistic children reported struggling to settle in and those with WS reported higher incidences of bullying. Secondly, although there was no change in anxiety levels over time, most participants showed clinical anxiety both pre‐ and post‐school transition. In addition, autistic and WS children showed the highest levels of anxiety at both time points. Nevertheless, children with WS were more anxious than autistic children. Next, there were some key differences in the predictive factors for anxiety amongst the neurodivergent children. For autistic children and those with DS, internalising behaviours were associated with high anxiety for pre‐school transition period. It also appears that children with WS with higher levels of social challenges in post‐school transition show higher anxiety.

The current findings suggest that school transition is a time of high anxiety for some neurodivergent children, who could benefit from additional support, especially mental wellbeing support, during their school transition. For example, support could focus on managing bullying and helping students settle in. In addition, the findings show that individual differences exist, and different factors may drive anxiety during transition to secondary school. As such, interventions should be tailored to the child with a focus on social and emotional support. Although further research is needed to understand the factors that could contribute to children with WS’ anxiety and those with DS during times of transition, the findings from the current study help shape the direction of future research.

## AUTHOR CONTRIBUTIONS

**Vassilis Sideropoulos**: Data curation; formal analysis; resources; software; validation; visualization; writing – original draft; writing – review & editing. **Olympia Palikara**: Conceptualization; data curation; formal analysis; funding acquisition; investigation; methodology; project administration; resources; software; supervision; validation; visualization; writing – original draft; writing – review & editing. **Elizabeth Burchell**: Data curation; writing – review & editing. **Maria Ashworth**: Data curation; writing – review & editing. **Jo Van Herwegen**: Conceptualization; data curation; formal analysis; investigation; methodology; project administration; resources; software; supervision; validation; visualization; writing – original draft; writing – review & editing.

## CONFLICT OF INTEREST STATEMENT

The authors have declared no competing or potential conflicts of interest.

## ETHICAL CONSIDERATIONS

Ethical approval for the study was obtained from the Ethics Committee of Kingston University prior to the data collection (Ethics REF: 171816). All participants (parents and neurodivergent children) provided online consent to participate in the study.

## Supporting information

Supporting Information S1

## Data Availability

The data and material that support the findings of this study are available upon request.
