# Supplementary material for: Anxiety during transition from primary to secondary schools in neurodivergent children
Source: JCPP Adv. 2024 Aug 13;5(2):e12262. doi: 10.1002/jcv2.12262 (PMC12159320; doi:10.1002/jcv2.12262)
Supplement: Supplementary file 1 — Supporting Information S1 [file JCV2-5-e12262-s001.docx]

**Supplementary Information 1**

**Cognitive and Behavioural Measures**

Multiple one-way analyses of variances (ANOVAs) were conducted. First, four one-way analyses of variances (ANOVAs) were conducted to compare (1) children’s cognitive abilities (BPVS and RCPM scores combined), (2) children’s SRS scores and (3) Non-adaptive Behaviour between the three neurodiverse groups. Next, a 2 (Timepoints) X 2(Internalising/Externalising) X 3 (Groups) Repeated Measures ANOVA was conducted to compare scores over time for the internalising and externalising sub-scales of SDQ.

**Cognitive Abilities**

Α one-way ANOVA for the newly computed variable Cognitive Abilities showed a group difference *F(*2, 56) = 57.311, *p* <.001, η_p_^2^ = .672. Table S1 presents the group differences.

| **Table S1 - Cognitive -Abilities - Post Hoc Comparisons - Group** | | | | | | | | | | | |
| --- | --- | --- | --- | --- | --- | --- | --- | --- | --- | --- | --- |
|  | |  | | **Mean Difference** | | **SE** | | **t** | | **p_bonf_** | |
| Autism |  | DS |  | 80.733 |  | 7.930 |  | 10.181 |  | < .001 |  |
|  |  | WS |  | 62.633 |  | 8.621 |  | 7.265 |  | < .001 |  |
| DS |  | WS |  | -18.100 |  | 8.946 |  | -2.023 |  | 0.143 |  |
|  | | | | | | | | | | | |
| *Note.*  P-value adjusted for comparing a family of 3 | | | | | | | | | | | |

**Social Responsiveness Scale**

Next, a one-way ANOVA compared SRS scores between the neurodiverse groups. There was a significant difference between groups, *F(*2, 58) = 4.200, *p* = .02, η_p_^2^ = .13. A Bonferroni post-hoc test showed that SRS scores in the DS group were significantly lower than scores in the autism (*p* = .03) group. There were no significant differences in SRS scores between the autism and WS groups (*p =* 1), nor the DS and WS groups (*p* = .08). Table S2 presents the post-hoc analysis.

| **Table S2 - SRS - Post Hoc Comparisons - Group** | | | | | | | | | | | | | | | |
| --- | --- | --- | --- | --- | --- | --- | --- | --- | --- | --- | --- | --- | --- | --- | --- |
|  | | | | | | **95% CI for Mean Difference** | | | |  | | | | | |
|  | |  | | **Mean Difference** | | **Lower** | | **Upper** | | **SE** | | **t** | | **p_bonf_** | |
| Autism |  | DS |  | 19.16 |  | 1.85 |  | 36.46 |  | 7.19 |  | 2.66 |  | 0.03 | * |
|  |  | WS |  | 0.48 |  | -18.61 |  | 19.57 |  | 7.94 |  | 0.06 |  | 1.00 |  |
| DS |  | WS |  | -18.68 |  | -38.44 |  | 1.08 |  | 8.22 |  | -2.27 |  | 0.08 |  |
|  | | | | | | | | | | | | | | | |
| * p < .05 | | | | | | | | | | | | | | | |
| *Note.*  P-value and confidence intervals adjusted for comparing a family of 3 estimates (confidence intervals corrected using the tukey method). | | | | | | | | | | | | | | | |

**Non-adaptive Behaviours**

A set of Repeated Measures ANOVAs were computed for the non-adaptive behaviour scores (VABS) and the internalising and externalising behaviours (SDQ) as both of these measures were taken at both T1 and T2. For the non-adaptive behaviour scores, there was no time effect *F(*1, 58) = 2.474, *p* = .121, η_p_^2^ = .007; no group effect *F(*2, 58) = 2.120, *p* = .129, η_p_^2^ = .0056. No interaction between Time and Group; *F(*2, 58) = 1.408, *p* = .253, η_p_^2^ = .008. Table S3 presents the post-hoc analysis.

| **Table S3 - Non-adaptive Behaviours - Post Hoc Comparisons - Group** | | | | | | | | | | | |
| --- | --- | --- | --- | --- | --- | --- | --- | --- | --- | --- | --- |
|  | |  | | **Mean Difference** | | **SE** | | **t** | | **p_bonf_** | |
| Autism |  | DS |  | 0.891 |  | 0.478 |  | 1.864 |  | 0.202 |  |
|  |  | WS |  | -0.013 |  | 0.528 |  | -0.025 |  | 1.000 |  |
| DS |  | WS |  | -0.905 |  | 0.546 |  | -1.656 |  | 0.309 |  |
|  | | | | | | | | | | | |
| Note.  P-value adjusted for comparing a family of 3 | | | | | | | | | | | |
| Note.  Results are averaged over the levels of: Time | | | | | | | | | | | |

**Internalising and Externalising**

Finally, for the internalising and externalising behaviours, the model revealed a group effect; *F(*2, 58) = 23.545, *p* <.001, η_p_^2^ = .278 and a Time X Group interaction effect; *F(*6, 174) = 5.341, *p* < .001, η_p_^2^ = .058 but no Time effect; *F(*3, 176) = 1.669, *p* = .175, η_p_^2^ = .009. Post-hoc Table S4 and S5 presents the detailed analysis.

| **Table S4 - Internalising - Post Hoc Comparisons - Group** |
| --- |

| **Post Hoc Comparisons - Group** | | | | | | | | | | | |
| --- | --- | --- | --- | --- | --- | --- | --- | --- | --- | --- | --- |
|  | |  | | **Mean Difference** | | **SE** | | **t** | | **p_bonf_** | |
| Autism |  | DS |  | 4.360 |  | 0.672 |  | 6.489 |  | < .001 |  |
|  |  | WS |  | 0.483 |  | 0.741 |  | 0.652 |  | 1.000 |  |
| DS |  | WS |  | -3.876 |  | 0.767 |  | -5.052 |  | < .001 |  |
|  | | | | | | | | | | | |
| Note.  P-value adjusted for comparing a family of 3 | | | | | | | | | | | |
| Note.  Results are averaged over the levels of: Time | | | | | | | | | | | |

| **Table S5 - Post Hoc Comparisons - Group ✻ Time** | | | | | | | | | | | |
| --- | --- | --- | --- | --- | --- | --- | --- | --- | --- | --- | --- |
|  | |  | | **Mean Difference** | | **SE** | | **t** | | **p_bonf_** | |
| Autism, Internalising T1 |  | DS, Internalising T1 |  | 6.686 |  | 0.930 |  | 7.192 |  | < .001 |  |
|  |  | WS, Internalising T1 |  | 2.133 |  | 1.026 |  | 2.080 |  | 1.000 |  |
|  |  | Autism, Internalising T2 |  | 0.880 |  | 0.709 |  | 1.241 |  | 1.000 |  |
|  |  | DS, Internalising T2 |  | 6.924 |  | 0.930 |  | 7.448 |  | < .001 |  |
|  |  | WS, Internalising T2 |  | 1.733 |  | 1.026 |  | 1.690 |  | 1.000 |  |
|  |  | Autism, Externalising T1 |  | 1.400 |  | 0.709 |  | 1.975 |  | 1.000 |  |
|  |  | DS, Externalising T1 |  | 3.781 |  | 0.930 |  | 4.067 |  | 0.005 |  |
|  |  | WS, Externalising T1 |  | 1.533 |  | 1.026 |  | 1.495 |  | 1.000 |  |
|  |  | Autism, Externalising T2 |  | 2.120 |  | 0.709 |  | 2.990 |  | 0.211 |  |
|  |  | DS, Externalising T2 |  | 4.448 |  | 0.930 |  | 4.784 |  | < .001 |  |
|  |  | WS, Externalising T2 |  | 0.933 |  | 1.026 |  | 0.910 |  | 1.000 |  |
| DS, Internalising T1 |  | WS, Internalising T1 |  | -4.552 |  | 1.062 |  | -4.288 |  | 0.002 |  |
|  |  | Autism, Internalising T2 |  | -5.806 |  | 0.930 |  | -6.245 |  | < .001 |  |
|  |  | DS, Internalising T2 |  | 0.238 |  | 0.774 |  | 0.308 |  | 1.000 |  |
|  |  | WS, Internalising T2 |  | -4.952 |  | 1.062 |  | -4.664 |  | < .001 |  |
|  |  | Autism, Externalising T1 |  | -5.286 |  | 0.930 |  | -5.686 |  | < .001 |  |
|  |  | DS, Externalising T1 |  | -2.905 |  | 0.774 |  | -3.755 |  | 0.016 |  |
|  |  | WS, Externalising T1 |  | -5.152 |  | 1.062 |  | -4.853 |  | < .001 |  |
|  |  | Autism, Externalising T2 |  | -4.566 |  | 0.930 |  | -4.911 |  | < .001 |  |
|  |  | DS, Externalising T2 |  | -2.238 |  | 0.774 |  | -2.893 |  | 0.284 |  |
|  |  | WS, Externalising T2 |  | -5.752 |  | 1.062 |  | -5.418 |  | < .001 |  |
| WS, Internalising T1 |  | Autism, Internalising T2 |  | -1.253 |  | 1.026 |  | -1.222 |  | 1.000 |  |
|  |  | DS, Internalising T2 |  | 4.790 |  | 1.062 |  | 4.512 |  | < .001 |  |
|  |  | WS, Internalising T2 |  | -0.400 |  | 0.915 |  | -0.437 |  | 1.000 |  |
|  |  | Autism, Externalising T1 |  | -0.733 |  | 1.026 |  | -0.715 |  | 1.000 |  |
|  |  | DS, Externalising T1 |  | 1.648 |  | 1.062 |  | 1.552 |  | 1.000 |  |
|  |  | WS, Externalising T1 |  | -0.600 |  | 0.915 |  | -0.656 |  | 1.000 |  |
|  |  | Autism, Externalising T2 |  | -0.013 |  | 1.026 |  | -0.013 |  | 1.000 |  |
|  |  | DS, Externalising T2 |  | 2.314 |  | 1.062 |  | 2.180 |  | 1.000 |  |
|  |  | WS, Externalising T2 |  | -1.200 |  | 0.915 |  | -1.311 |  | 1.000 |  |
| Autism, Internalising T2 |  | DS, Internalising T2 |  | 6.044 |  | 0.930 |  | 6.501 |  | < .001 |  |
|  |  | WS, Internalising T2 |  | 0.853 |  | 1.026 |  | 0.832 |  | 1.000 |  |
|  |  | Autism, Externalising T1 |  | 0.520 |  | 0.709 |  | 0.733 |  | 1.000 |  |
|  |  | DS, Externalising T1 |  | 2.901 |  | 0.930 |  | 3.120 |  | 0.141 |  |
|  |  | WS, Externalising T1 |  | 0.653 |  | 1.026 |  | 0.637 |  | 1.000 |  |
|  |  | Autism, Externalising T2 |  | 1.240 |  | 0.709 |  | 1.749 |  | 1.000 |  |
|  |  | DS, Externalising T2 |  | 3.568 |  | 0.930 |  | 3.838 |  | 0.012 |  |
|  |  | WS, Externalising T2 |  | 0.053 |  | 1.026 |  | 0.052 |  | 1.000 |  |
| DS, Internalising T2 |  | WS, Internalising T2 |  | -5.190 |  | 1.062 |  | -4.889 |  | < .001 |  |
|  |  | Autism, Externalising T1 |  | -5.524 |  | 0.930 |  | -5.942 |  | < .001 |  |
|  |  | DS, Externalising T1 |  | -3.143 |  | 0.774 |  | -4.063 |  | 0.005 |  |
|  |  | WS, Externalising T1 |  | -5.390 |  | 1.062 |  | -5.077 |  | < .001 |  |
|  |  | Autism, Externalising T2 |  | -4.804 |  | 0.930 |  | -5.167 |  | < .001 |  |
|  |  | DS, Externalising T2 |  | -2.476 |  | 0.774 |  | -3.201 |  | 0.107 |  |
|  |  | WS, Externalising T2 |  | -5.990 |  | 1.062 |  | -5.642 |  | < .001 |  |
| WS, Internalising T2 |  | Autism, Externalising T1 |  | -0.333 |  | 1.026 |  | -0.325 |  | 1.000 |  |
|  |  | DS, Externalising T1 |  | 2.048 |  | 1.062 |  | 1.929 |  | 1.000 |  |
|  |  | WS, Externalising T1 |  | -0.200 |  | 0.915 |  | -0.219 |  | 1.000 |  |
|  |  | Autism, Externalising T2 |  | 0.387 |  | 1.026 |  | 0.377 |  | 1.000 |  |
|  |  | DS, Externalising T2 |  | 2.714 |  | 1.062 |  | 2.556 |  | 0.757 |  |
|  |  | WS, Externalising T2 |  | -0.800 |  | 0.915 |  | -0.874 |  | 1.000 |  |
| Autism, Externalising T1 |  | DS, Externalising T1 |  | 2.381 |  | 0.930 |  | 2.561 |  | 0.747 |  |
|  |  | WS, Externalising T1 |  | 0.133 |  | 1.026 |  | 0.130 |  | 1.000 |  |
|  |  | Autism, Externalising T2 |  | 0.720 |  | 0.709 |  | 1.016 |  | 1.000 |  |
|  |  | DS, Externalising T2 |  | 3.048 |  | 0.930 |  | 3.278 |  | 0.084 |  |
|  |  | WS, Externalising T2 |  | -0.467 |  | 1.026 |  | -0.455 |  | 1.000 |  |
| DS, Externalising T1 |  | WS, Externalising T1 |  | -2.248 |  | 1.062 |  | -2.117 |  | 1.000 |  |
|  |  | Autism, Externalising T2 |  | -1.661 |  | 0.930 |  | -1.787 |  | 1.000 |  |
|  |  | DS, Externalising T2 |  | 0.667 |  | 0.774 |  | 0.862 |  | 1.000 |  |
|  |  | WS, Externalising T2 |  | -2.848 |  | 1.062 |  | -2.682 |  | 0.532 |  |
| WS, Externalising T1 |  | Autism, Externalising T2 |  | 0.587 |  | 1.026 |  | 0.572 |  | 1.000 |  |
|  |  | DS, Externalising T2 |  | 2.914 |  | 1.062 |  | 2.745 |  | 0.444 |  |
|  |  | WS, Externalising T2 |  | -0.600 |  | 0.915 |  | -0.656 |  | 1.000 |  |
| Autism, Externalising T2 |  | DS, Externalising T2 |  | 2.328 |  | 0.930 |  | 2.504 |  | 0.875 |  |
|  |  | WS, Externalising T2 |  | -1.187 |  | 1.026 |  | -1.157 |  | 1.000 |  |
| DS, Externalising T2 |  | WS, Externalising T2 |  | -3.514 |  | 1.062 |  | -3.310 |  | 0.076 |  |
|  | | | | | | | | | | | |
| Note.  P-value adjusted for comparing a family of 66 | | | | | | | | | | | |
